# Mathematical modelling with experimental validation of viscoelastic properties in non-Newtonian fluids

**DOI:** 10.1098/rsta.2019.0284

**Published:** 2020-05-11

**Authors:** C. M. Ionescu, I. R. Birs, D. Copot, C. I. Muresan, R. Caponetto

**Affiliations:** 1 Ghent University, Department of Electromechanical, Systems and Metal Engineering, Research laboratory on Dynamical Systems and Control, Tech Lane Science Park 125, 9052 Ghent, Belgium; 2 Technical University of Cluj Napoca, Department of Automatic Control, Memorandumului street 28, Cluj, Romania; 3 Flanders Make, EEDT - Decision and Control Group, Tech Lane Science Park 131, 9052 Ghent, Belgium; 4 Universita’ degli Studi di Catania, Department of Engineering, Electric, Electronic and Informatics, Viale Andrea Doria 6, 95125 Catania, Italy

**Keywords:** non-Newtonian fluids, viscoelasticity, fractional-order impedance model, frequency response, genetic algorithm

## Abstract

The paper proposes a mathematical framework for the use of fractional-order impedance models to capture fluid mechanics properties in frequency-domain experimental datasets. An overview of non-Newtonian (NN) fluid classification is given as to motivate the use of fractional-order models as natural solutions to capture fluid dynamics. Four classes of fluids are tested: oil, sugar, detergent and liquid soap. Three nonlinear identification methods are used to fit the model: nonlinear least squares, genetic algorithms and particle swarm optimization. The model identification results obtained from experimental datasets suggest the proposed model is useful to characterize various degree of viscoelasticity in NN fluids. The advantage of the proposed model is that it is compact, while capturing the fluid properties and can be identified in real-time for further use in prediction or control applications.

This article is part of the theme issue ‘Advanced materials modelling via fractional calculus: challenges and perspectives’.

## 1. Introduction

Most properties of non-Newtonian (NN) fluids overlap with that of viscoelastic materials, such as polymers, lung tissue, gel-like substances, rubber, etc. [1,2]. Specific properties as memory, creep and shear stress do not follow classical Newton’s Law of Viscosity and has been proven to be well characterized by combinations of power-law and exponential functions [3,4]. These are non-rational expressions of combined nonlinear effects in material creep and strain which have been well characterized by the nonlinear Mittag–Leffler function [5–7]. In particular, they represent a generalization from integer-order differential equations to fractional-order differential equations. Such generalized order equations are mathematical tools emerging from fractional calculus and successfully introduced in engineering, medical and many other applications areas [3,8,9].

The great potential of fractional-order impedance models (FOIMs) for capturing natural properties of materials in a variety of disciplines has been long recognized and established experimentally [10–13]. Applications in medicine and biology are most prevalent as these dynamical systems feature core properties such as multi-scale dynamics, diffusion, viscoelasticity and relaxation [8,14–16]. The same properties are often used to model dynamics in the areas of geology, manufacturing, food industry and chemical products. The list of their applicability is vast and summarized in several excellent surveys [3]. The prevalence of these properties is much increased in NN fluids and soft materials. As such, one can identify classes of NN fluids in each of the above-mentioned application areas.

Fractional-order systems have been recently applied in modelling NN fluid. In [17], the authors propose a fraction seepage model of a NN fluid. The non-local characteristic of the fluid has been modelled in the case of a porous media. In the paper, the authors make an attempt to relate the fractional order of the model with the fractal dimension of media tortuosity. A further application can be found in [18], where the authors in the case of a steady pipe flow, describe, via fractional derivative models, the flow of NN fluid driven by spatially non-local velocity. A more specific fractional system, the extension of the fractional-order Maxwell model, has been applied in [19] to incorporate a relaxation process with NN viscosity behaviour. The main contribution of the fractional-order systems approach has been noted in the capability of factional calculus to describe the effect of non-localities as well the memory effect. Furthermore, the integer-order exponents of the integro-differential equations can be used as further optimization parameters in measurement fitting.

A more common approach in modelling and simulating NN behaviour is based on the numerical calculus via computational fluid dynamics (CFD). In this case, dedicated software such as Ansys Fluent, Open Foam, SIMSCALE, SimFlow, to mention just a few, are used. In this case, effective applications can be found in different areas. In [20], Newtonian and NN blood viscosity models have been considered in simulating the flow in atherosclerotic coronary arteries; in [21] the authors, in order to realize a continuous way of measuring and monitoring drilling fluid properties, simulate a NN fluid that is best described by a Yield-power-law (YPL) rheological model. Numerical methods and time complex time-domain models using power law models and fractional derivatives have been proposed in [22–24], providing excellent simulation analysis tools. The memory property of such fluids was also captured with variations of the fractional order in time [22]. Such in-depth theoretical analysis is a solid basis and motivation for using the FOIMs.

The term FOIM was coined some decades ago in an application of modelling respiratory tissue properties such as tissue compliance as a function of anatomical and structural changes in respiratory disorders [15]. It has since then used in many applications, such as tissue modelling, drug diffusion and blood viscosity [5]. The high versatility of FOIMs stands in their combination of general-order integrators and differentiators. The fractional orders are usually limited between physical values capturing properties defined by classical (Newtonian) theory of fluids or materials.

The representation of FOIMs in the frequency domain greatly simplifies their applicability to experimental data. The collection of data is time based, but suitably transformed into equivalent complex or polar coordinates. Dynamic response methods are applied to obtain the material reaction to an input signal suitably designed. Although there are time-based models defined for capturing material properties, these require a high computational complexity and a careful choice of time-based definitions [25].

A great advantage of FOIMs expressed in Laplace and their equivalent frequency-domain forms is their capability to capture in a compact form complex nonlinear properties and have these identified in a real-time context. This was previously shown in modelling memory effects in blood [5] and in designing a closed-loop control of suspended objects in a blood-like varying context of viscoelastic fluid properties [26]. Beyond the specific application, this paper proposes to use a mathematical framework of FOIMs to capture and link the model parameter structure to the existence of specific properties in NN fluids. The novelty of this approach consists in the justification of using FOIMs, as well as the systematic analysis of the FOIM structure versus fluid properties. Novel is also the experimental protocol designed and applied to a series of fluid classes (oil, sugar, detergent and liquid soap). An additional original element of this research consists in the presentation and validation of several variations of FOIMs on the dataset. Finally, the set of recommendations concerning the future use of FOIMs in capturing material properties is also novel.

The paper is organized as follows. The next section introduces the properties of NN fluids and provides the necessary motivation for using FOIMs. The third section provides an overview of the various FOIM structures and identification algorithms. The fourth section summarizes the results, followed by the fifth section with discussion and recommendations. The main outcome of this work is summarized in the conclusion.

## 2. Non-Newtonian fluids classification

In this section, we familiarize the reader with the minimal textbook background in properties of NN fluids and soft materials [1,2]. This information also provides the necessary motivation for using FOIMs in characterizing such material properties.

**Figure 1.**
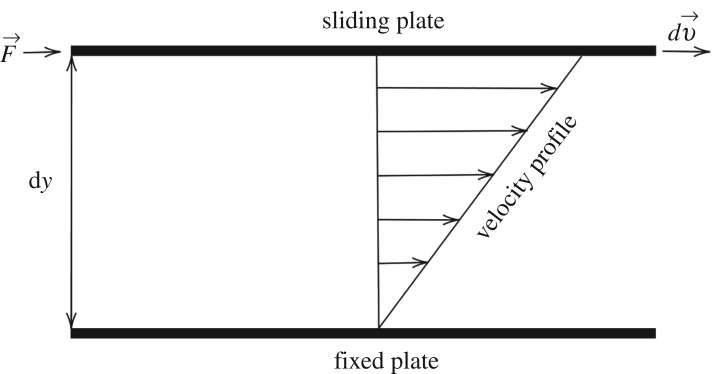
Schematic of unidirectional shearing flow.

Consider the infinitesimal volume fluid element made by the overlapping of infinitesimal thickness layer. If a very thin layer of Newtonian fluid is contained within two plates, as in figure 1, it is possible to observe the linearity of the fluid’s velocity profile as a consequence of a shearing stress **F** applied to the moving plate [2]. In this linear context, the external force is balanced by an internal frictional force in the fluid arising from its viscosity. In other words, the fluid is subjected to a uniform strain rate $\dot{\gamma }$: the governing equation that relates the shear stress *τ*_yx_, pressure-like, and the shear rate $\dot{\gamma_{\mathrm{yx}}}$, or strain rate, is

2.1 $$\tau_{\mathrm{yx}}=-\eta \;\dot{\gamma_{\mathrm{yx}}},$$

also known as *Newton’s Law of Fluids*. The subscript yx is used to underline the direction of the stress and strain in the fluid. The minus sign on the right-hand side of this relation suggests that the shear stress is a resisting force. Looking at (2.1) the similarity with Hook’s Law for a solid can be noticed, with the difference that we are referring all to the share rate $\dot{\gamma_{\mathrm{yx}}}$. Moreover, the relation is linear and the constant coefficient *η* is called viscosity of the fluid. This value is well determined for a great variety of Newtonian fluids, although it may vary with temperature and pressure of the fluids, hence changing the overall physical properties of the fluid. All gases, as well as most common liquids like water, oils, hydrocarbon and also metals, in liquid form, are examples of Newtonian fluids.

By contrast, a fluid is called NN when it does not respect Newton’s Law. This means that the relation between shear stress and shear rate is no longer linear, i.e. the viscosity varies in time, dependent on conditions such as

— flow conditions, e.g. flow geometry,
— shear stress applied to the fluid,
— shear rate developed within the fluid,
— time of shearing stress applied,
— kinematic history of the sample, etc.

**Figure 2.**
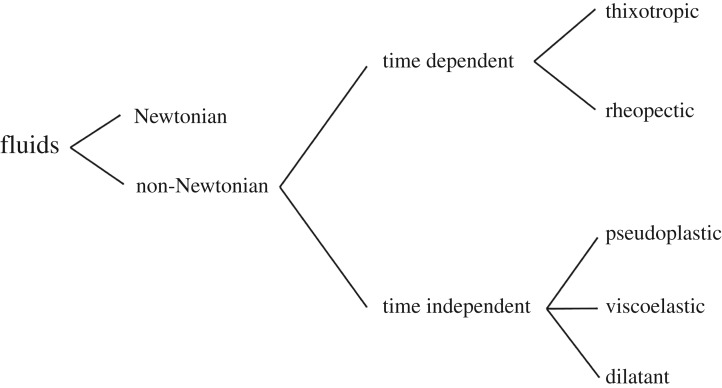
Classification tree of non-Newtonian fluids.

A classification of NN fluids in different categories can be made based on the main origin of viscosity changes, as given in figure 2. Note however that this classification is quite arbitrary, because most real materials often display a combination of two or more properties. Nevertheless, in most cases, it is possible to identify the dominating NN feature and to use it as the basis for subsequent process engineering calculations.

### (a) Time-independent properties

Fluids whose flow properties are independent of the duration of shearing may be described as

2.2 $$\tau_{\mathrm{yx}}=f(\dot{\gamma_{\mathrm{yx}}})\;\mathrm{or}\;\mathrm{vice-versa}\;\dot{\gamma_{\mathrm{yx}}}=f^{-1}(\tau_{\mathrm{yx}}).$$

This equation implies that the rate of shear, at any point within the sheared fluid, is determined solely by the current value of the shear stress at that point, or vice versa. Depending upon the form of (2.2), these fluids may be further subdivided into three different types:

(i) shear-thinning or pseudoplastic,
(ii) viscoplastics, including Bingham plastics,
(iii) shear-thickening or dilatant.

#### (i) Pseudoplastic or shear-thinning fluids

A great part of commonly known fluids belong to this family: nail polish, whipped cream, ketchup, molasses, syrups, paper pulp in water, latex paint, ice, some silicone oils, sand in water and some silicone coatings. These kinds of fluids have an apparent viscosity, i.e. the ratio between shear stress and rate, which decreases with the increment of the shear rate. Most pseudoplastic fluids are piecewise linear and the following limit variables can be introduced:

2.3*a* $$\lim_{\dot{\gamma_{\mathrm{yx}}}\to 0}\frac{\tau_{\mathrm{yx}}}{\dot{\gamma_{\mathrm{yx}}}}=\eta_{0}\;\text{zero shear viscosity}$$

and

2.3*b* $$\lim_{\dot{\gamma_{\mathrm{yx}}}\to \infty }\frac{\tau_{\mathrm{yx}}}{\dot{\gamma_{\mathrm{yx}}}}=\eta_{\infty }\;\text{infinite shear viscosity}.$$

**Figure 3.**
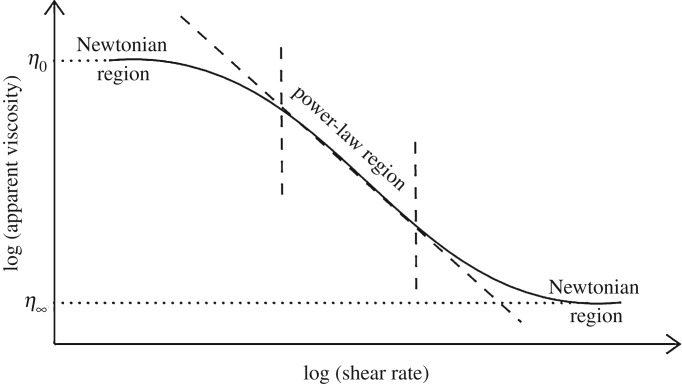
Qualitative representation of the apparent viscosity behaviour for a shear-thinning fluid.

In figure 3, we observe that the central region of the curve is piecewise linear. Taking into account that the plot is in logarithmic scale, this behaviour can be well modelled as a power-law relation

2.4 $$\tau_{\mathrm{yx}}=m{\left(\dot{\gamma_{\mathrm{yx}}}\right)}^{n},$$

or by using the (2.1) in (2.4) and resolving with respect to the viscosity one obtains

2.5 $$\eta =m{\left(|\dot{\gamma_{\mathrm{yx}}}|\right)}^{n-1},$$

which is also known as the *Ostwald–De Waele model* for pseudoplastic fluid [27]. The two model parameters *n* and *m* are called, respectively, the power-law index and the fluid consistency coefficient. For a Newtonian fluid *n* = 1, while for a pseudoplastic substance *n* < 1. The lower the value of the power-law index, the greater is the degree of shear-thinning. Admittedly, (2.5) provides the simplest description of shear-thinning behaviour, but it also has a number of limitations. Another model is based on the assumption that the shear-thinning behaviour is caused by the formation and breakdown of ‘structural linkages or units’, also observed in polymers and lung tissue [4]. For one-dimensional steady shearing, we have

2.6 $$\frac{\eta -\eta_{\infty }}{\eta_{0}-\eta_{\infty }}=\frac{1}{1+{\left(\lambda \dot{\gamma_{\mathrm{yx}}}\right)}^{2/3}},$$

where *η*_0_ and *η*_∞_, respectively, are the zero and infinite shear viscosities while λ is a constant with units of time. This formula was reported to satisfactorily fit the shear stress-shear rate data for a wide variety of pseudoplastic systems. Moreover, this model is the clear evidence of how the theory of fractional-order systems can be more suitable to reproduce this kind of behaviour, rather than integer-order approximation.

#### (ii) Visco-plastic fluids

This class of materials is characterized by the existence of a minimal yield stress *τ*_0_ that must be exceeded before deformation (or flow) occurs. One can explain this behaviour by postulating that the substance at rest consists of a three-dimensional structure of sufficient rigidity or strength to resist any external stress less than *τ*_0_. For stress levels greater than *τ*_0_, the structure loosens, and the material behaves as a viscous fluid. A fluid with a linear flow curve for $|\tau_{\mathrm{yx}}|>\tau_{0}$ is called a Bingham plastic fluid and is characterized by a constant value of plastic viscosity [9].

Among the many mathematical equations to model this kind of flow behaviour, the simplest and perhaps the most widely used can be written as

2.7 $$\begin{array}{rl} & \tau_{\mathrm{yx}}=\tau_{0}^{B}+\eta \;\dot{\gamma_{\mathrm{yx}}}\;\mathrm{if}\;\tau_{\mathrm{yx}}>\tau_{0}^{B}\\ \text{and}\; & \dot{\gamma_{\mathrm{yx}}}=0\;\mathrm{if}\;\tau_{\mathrm{yx}}\leq \tau_{0}^{B}, \end{array}\}$$

where $\tau_{0}^{B}$ is the Bingham yield stress and *η*_B_ is the plastic viscosity. Another model for visco-plastic fluids is known as the *Herschel–Bulkley* model [28], written for a simple shear flow as

2.8 $$\begin{array}{rl} & \tau_{\mathrm{yx}}=\tau_{0}^{H}+m\;{\left(\dot{\gamma_{\mathrm{yx}}}\right)}^{n}\;\mathrm{if}\;\tau_{\mathrm{yx}}>\tau_{0}^{H}\\ \text{and} & \dot{\gamma_{\mathrm{yx}}}=0\;\mathrm{if}\;\tau_{\mathrm{yx}}\leq \tau_{0}^{H}. \end{array}\}$$

This formula can be seen as a generalization of the simple Bingham model (2.7), in which the linear shear rate dependence has been replaced by a power-law behaviour. This model is broadly used to model blood viscosity [5] and muddy clay modelling applications [29].

#### (iii) Dilatant or shear-thickening fluids

These materials, also known as dilatant materials, are similar to shear-thinning materials in that they show no yield stress, but their apparent viscosity increases with increasing shear rate. This type of behaviour is encountered in concentrated suspensions of solids, and can be qualitatively explained as follows. When a suspension is at rest, the gap between one particle and another is the minimum and the liquid present is just sufficient to fill the void spaces. At low shear rates, the liquid lubricates the motion of one particle past another, and the resulting stresses are consequently low. At high shear rates, on the other hand, the dense packing of solids breaks down and the material expands or dilates slightly causing an increase in the gap, and thus the amount of liquid available is no longer sufficient to lubricate the solid motion of one particle past another and the resulting solid–solid friction causes the stresses to increase rapidly, which, in turn, causes an increase in the apparent viscosity. In the past decades, experimental data suggest that the apparent viscosity–shear rate curves often result in a linear behaviour on log-log coordinates over a limited shear rate range of interest, and thus the power-law model (2.5) may be used with *n* > 1 in this case.

**Figure 4.**
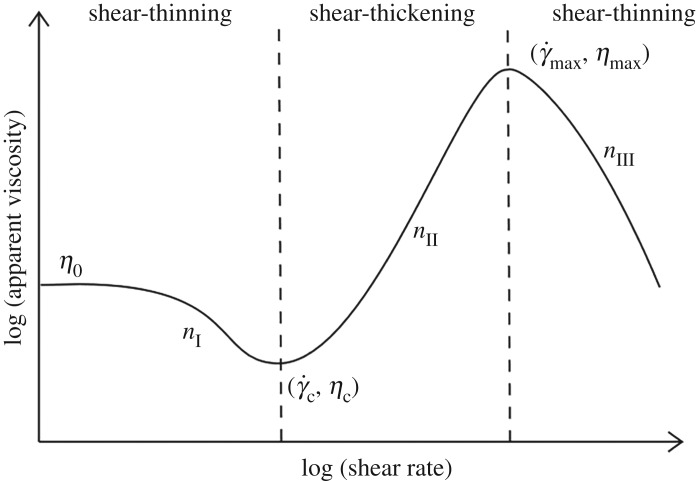
Qualitative viscosity curve for a shear-thickening fluid.

The entire behaviour of typical shear-thickening fluids, as given in figure 4, gives evidence of three distinct zones. Two of them, for very low and very high shear rate, respectively, are regions in which the fluid shows shear-thinning characteristics, i.e. the viscosity decreases with increasing shear rates. However, for mid-range shear rate, the liquid behaves like shear-thickening, resulting in increasing viscosity with shear rate. This region is linear in the log-log plot, as mentioned earlier. The model has been revised taking into account the three different regions:

2.9 $$\eta =\left\{ \begin{array}{ll} \eta_{\text{c}}+\frac{\eta_{0}-\eta_{\text{c}}}{1+{\left[K_{\text{I}}({\dot{\gamma }}^{2}/(\dot{\gamma }-{\dot{\gamma }}_{\text{c}}))\right]}^{n_{\text{I}}}} & \mathrm{for}\;\;\dot{\gamma }\leq {\dot{\gamma }}_{\text{c}}\\ \eta_{\text{max}}+\frac{\eta_{\text{c}}-\eta_{\text{max}}}{1+{\left[K_{\text{II}}((\dot{\gamma }-{\dot{\gamma }}_{\text{c}})/(\dot{\gamma }-{\dot{\gamma }}_{\text{max}}))\dot{\gamma }\right]}^{n_{\text{II}}}} & \mathrm{for}\;\;{\dot{\gamma }}_{\text{c}}<\dot{\gamma }\leq {\dot{\gamma }}_{\text{max}}\\ \frac{\eta_{\text{max}}}{1+{\left[K_{\text{III}}(\dot{\gamma }-{\dot{\gamma }}_{\text{max}})\right]}^{n_{\text{III}}}} & \mathrm{for}\;\;\dot{\gamma }>{\dot{\gamma }}_{\text{max}}. \end{array}\right.$$

It must be noted that the three branches of (2.9) have a functional form based on that of the Cross model: the similarity can be found if (2.6) is rearranged by solving it with respect to *η*. Hence, the parameters appearing in (2.9) have the same dimensions and interpretation: *K*_I_, *K*_II_, *K*_III_ possess time dimension and are responsible for the transitions between the plateaus and the power-law, while the dimensionless exponents *n*_*i*_ are related to the slopes of the power-law regimes. Additionally, it is sufficient to calculate the right and left limit of the three branches to see that the function is continuous.

### (b) Time-dependent properties

For many industrial materials, as well as some common food materials, the viscosity depends on both shear rate and shear time. The most common example is honey: when it is sheared at a constant rate of shear, following a period of rest, its apparent viscosity gradually decreases as its internal ‘structure’ breaks down progressively. As the number of ‘structural linkages’ available for breaking down decreases, also the rate of variation drops towards zero. On the other hand, the rate at which the linkages can reform increases, and eventually a state of dynamic equilibrium is reached when the rates of build-up and breakdown linkages are equal. This type of fluid behaviour may be further divided into two categories: thixotropic and rheopectic or anti-thixotropic.

#### (i) Thixotropic fluids

**Figure 5.**
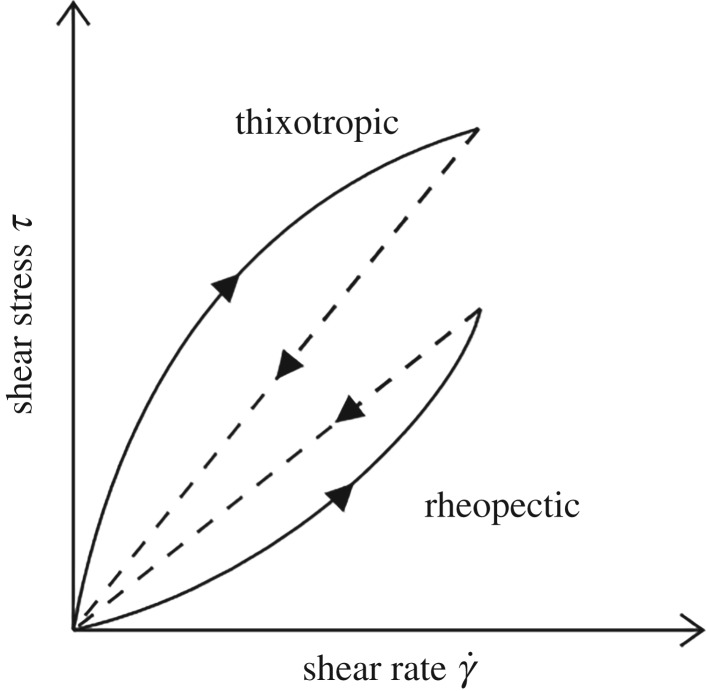
Qualitative shear stress-shear rate behaviour for thixotropic and rheopectic materials.

A material is said to exhibit thixotropy if its apparent viscosity (or shear stress) decreases with time when sheared at a constant rate of shear. If the flow curve is measured in a single experiment in which the shear rate is steadily increased at a constant rate from zero to a maximum value, and then decreased at the same rate to zero again, a hysteresis loop, as shown schematically in figure 5, is obtained. The height, the shape and the enclosed area of the loop depend on the kinematic parameters such as duration and rate of shearing, past deformation history of the material sample, etc.

In practice, thixotropic substances show very similar properties to the shear-thinning fluids with the difference that the former do not show constant viscosity if a fixed value shear rate is kept for a while.

Common examples are yogurt, peanut butter, honey, aqueous iron oxide gels, gelatin gels, carbon black suspension in molten tyre rubber, some drilling muds, many paints, many colloidal suspensions.

#### (ii) Rheopectic fluids

The relatively few systems for which the apparent viscosity increases with the duration of shearing are said to display rheopexy, or negative thixotropy. Again hysteresis effects are observed in the flow curve in figure 5, but in this case it is inverted compared to that for a thixotropic material. By analogy with thixotropy, rheopexy is associated with a gradual build-up of ‘structure’ as the fluid is sheared, though it is not certain whether an equilibrium will ever be reached. Thus, in a rheopectic material, the structure builds up by shear and it breaks down when the material is at rest. For this reason, it can be associated with the shear-thickening, or dilatant fluids.

Some examples of rheopectic fluids are synovial fluid, printer ink, gypsum paste.

To conclude, we have observed that the material properties can change as a function of various dynamic conditions, requiring several individual model structures. Power-law, exponential and combinations thereof are commonly shown to be dynamics which naturally require generalized models such as fractional-order models. Although time-domain fractional-order model representation exists, the remainder of this paper will deal with FOIMs represented in Laplace domain for frequency-domain identification.

## 3. Material and methods

### (a) Experimental set-up and measurement protocol

**Figure 6.**
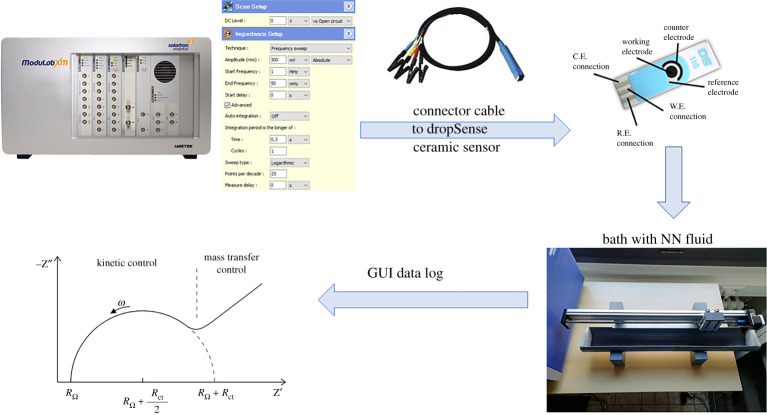
ModuLab XM measurement device with afferent instrumentation for experimental testing of various NN fluid impedance characteristics. The GUI data are a polar plot, with real and imaginary parts as a function of excited frequencies. (Online version in colour.)

The device depicted in figure 6 performs electrochemical impedance spectroscopy in fluid samples. The method behind is the classical transfer function analyser algorithm. The reference signal is applied by means of a *potentiostat* and then through the *frequency response analyser* the actual measurement of the impedance can be performed. The *Modulab XM* is a highly versatile electrochemical test system that measures the characteristics of a wide range of materials including organic/inorganic, specialized corrosion, electroplating and energy cells. Reference grade system components (potentiostat/galvanostat, frequency response analyser and optional high voltage amplifier) are combined in a single unit, avoiding the need for stacking and wiring separate units. The device communicates via an Ethernet link to an external PC, running XM-studio ECS software for control and monitoring purposes. The testing signal covers a range of frequencies from μHz to MHz.

The software XM-studio ECS provides the data logging in a graphical form as a polar plot, and in an Excel file as numerical values for further processing. The measurement options are listed below:

— DC level: 0 mV
— RMS amplitude: 300 mV
— starting frequency: 1 MHz
— ending frequency: 5 Hz
— integration period time: 0.3 s
— integration period cycles: 1
— sweep type: logarithmic
— points per decade: 25

### (b) Fractional-order impedance model

A commonly used FOIM for (biological) material characterization is the model given by

3.1 $$Z(s)=R+Ls^{\alpha }+\frac{1}{Cs^{\beta }},$$

as a function of the Laplace variable *s*, containing a scaling factor *R* and two fractional-order terms which denote low- and high-frequency dependency whose gains *L* and *C* are constants denoting gain in the slope of the constant phase intervals related to the fractional-order values (for details on derivation see for example [3,4,14]). This is a compact model, consisting of the minimal term to characterize variations in frequency response of combined increasing and decreasing monotonic values. This model has been shown to be the natural solution of materials with mechanical properties modelled by combinations of Maxwell, Kelvin and Voigt elements [4,30]. This implies also that the model structure and parameters may be suitable for detecting various degrees of viscoelasticity.

Looking at the model in (3.1) and considering the fact that the parameter *α* always has a negative value, then it is possible to make some calculation to rearrange the model as

3.2 $$Z(s)=\frac{Rs^{\alpha +\beta }+Ls^{\beta }+Ds^{\alpha }}{s^{\alpha +\beta }},\;-2<\alpha ,\beta <2,$$

with *D* = 1/*C*. Therefore, it is possible to note that for some materials the appropriate model may have a pole in the origin and two other fractional-order derivative. However, doing an electrical comparison, this impedance could correspond to a circuit which has one resistor and two fractional-order capacitors, also known as constant phase elements. Such a model is broadly used for dielectric materials and neural network transmission pathways [31].

The complete FOIM will take the form of (3.1) in frequency domain as

3.3 $$Z(j\omega )=R+L{\left(j\omega \right)}^{\alpha }+\frac{D}{{\left(j\omega \right)}^{\beta }},\;-2<\alpha ,\beta <2,$$

which is a five-parameter model. When optimization is involved, it may be useful to verify the equivalent real and imaginary parts of this model

3.4*a* $$R\{Z(j\omega )\}=R+L\omega^{\alpha }\cos(\frac{\alpha \pi }{2})+\frac{D}{\omega^{\beta }}\cos(\frac{\beta \pi }{2})$$

and

3.4*b* $$I\{Z(j\omega )\}=-[L\omega^{\alpha }\sin(\frac{\alpha \pi }{2})+\frac{D}{\omega^{\beta }}\sin(\frac{\beta \pi }{2})],$$

where one can see the real part is no longer constant with frequency as in the classical integer-order formulation, but it varies as a function of frequency.

In the remainder of this paper, the full model has been used to characterize the impedance data, assuming the variations in the parameter values will depend on the degree of viscoelasticity in the test sample.

### (c) Identification methods

#### (i) Nonlinear least-squares algorithm

The available nonlinear least-squares minimization algorithm from Matlab has been used to fit the FOIM to the test sample data. The initial values are randomly selected at the beginning of the optimization from the intervals of feasible region.

The function lsqnonlin was used for the following options:

— 1000 different calls;
— 500 maximum number of iteration per call;
— 1 × 10^−9^ as goal for the cost function minimum;
— 10 recurrent iterations to further optimize the solution.

#### (ii) Genetic algorithm

The genetic algorithm (GA) is a biologically inspired optimization algorithm [32]. It is a stochastic global search method that tries to mimic the process of natural biological evolution. This algorithm operates on a population of potential solutions applying the principle of survival of the fittest, ideally to produce increasingly better approximations towards an optimal solution. At each generation, a new set of approximations is created by the process of selecting individuals according to their level of fitness in the problem domain and breeding them together using operators borrowed from natural genetics. This process leads to the evolution of populations of individuals that are better suited to their environment than the individuals that they were created from, just as in a natural adaptation mechanism.

**Figure 7.**
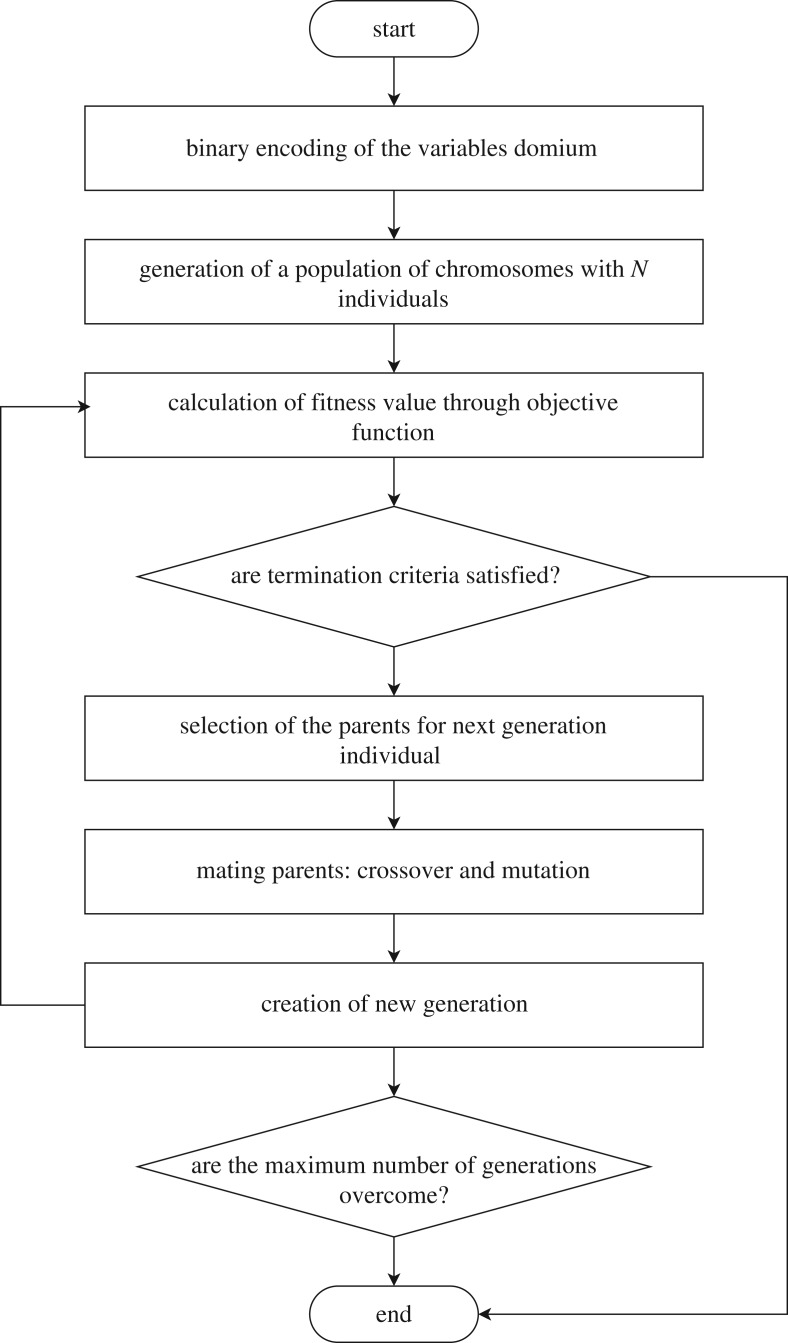
Flowchart of typical implementation of the genetic algorithm.

Figure 7 illustrates the flowchart of the GA, emphasizing the main steps. At the very beginning, the domain for each parameter to be found must be set properly. It is straightforward to understand that the larger the domain the more difficulties that the optimization process can have, especially if the problem is strongly nonlinear. Each of this range is then ‘discretized’ through a binary conversion, according to the specified number of bits. The binary elements of each range are called *chromosomes*. The binary number is called the *genotype* while the relative decimal, real number, represents the *phenotype*. Then, the first population is created by random selecting chromosomes and the size depends on the number of individuals, that is an option to set properly. The key role of the optimization process is played by the *objective function*: it can be seen as the cost function to be minimized and is the one which actually represents the problem to solve. Each chromosome is therefore converted to get the phenotype, and it is evaluated through the objective function: the value of the function for the given set of parameters is called *fitness value*. This allows one to asses the goodness of the chromosome determining its probability to be selected. In this version of the algorithm, the *roulette wheel* selection method is used: it means that the chromosomes selected to become the parents of the next generation individuals are chosen with a probability proportional to their fitness value. Note that the objective function has to be minimized such that the better the chromosome is, the lower the objective function value is: for this reason, the fitness value is usually its inverse.

After the selection, the chromosomes mate in couples and the son will have part of the genotype from both parents: this kind of process is called *crossover*. It represents the biological ability to evolve through generations inheriting good genes. Another process that can occur is called *mutation* and consists in the random change of only one-bit state in the genotype. This can be seen as a perturbation given to the solution, useful to escape from local minimums and converge towards the global minimum, if one exists. Each pair generates two sons and the probability that crossover and mutation happens are *P*_*c*_ and *P*_*m*_ established in the program, with *P*_*m*_ ≪ *P*_*c*_. Once the new population is generated, the process restart and continue until the stopping criteria is met, usually regarding a lower bound for the objective function and a maximum number of generation. An additional strategy purposes that the size of the offspring should not remain the same, and though another optional parameter is introduced called *generation gap*, representing the ratio between the new population and the old one. In this case, the GA is said to follow an elitist strategy.

The function ga has been implemented in Matlab with the following options:

— 10 independent calls of the algorithm;
— 1000 individuals per generation;
— 300 maximum number of generations;
— 90% of generation gap;
— 5 parameter to optimize, i.e. number of chromosomes;
— 10 bit to discretize the domain;
— limited range; and
— 1 × 10^−9^ as limit value for the cost function minimum.

#### (iii) Particle swarm optimization

The particle swarm optimization (PSO) concept was introduced in 1995, by simulating social behaviour of observed in animals or insects, e.g. bird flocking, fish schooling; afterwards the algorithm was simplified and it was observed to be performing optimization [33]. PSO is an evolutionary computation technique that optimizes a problem by iteratively trying to improve a candidate solution with regard to a given measure of quality.

It is inspired by the behaviours of swarms based on their movement and intelligence, which are seeking the most fertile feeding location. A swarm is a seemingly badly planned and disordered collection of moving individuals that tend to gather closely while each individual moves with random changes in direction. It uses a collection of particles that are part of a swarm moving around in the search space for finding the best solution to an optimization problem. The concept consists of changing the velocity (or accelerating) of each particle toward its *p*_best_ and the *g*_best_ position at each time step.

Each particle adjusts its own trajectory in an *n*-dimensional space, according to its own trajectory experience and the experience of other particles in the swarm. Each particle keeps track of the best position in the problem space, which it has reached so far. This value is called *p*_best_. Another best value called *g*_best_ is achieved by any particle associated with the best value found among all the particles.

**Figure 8.**
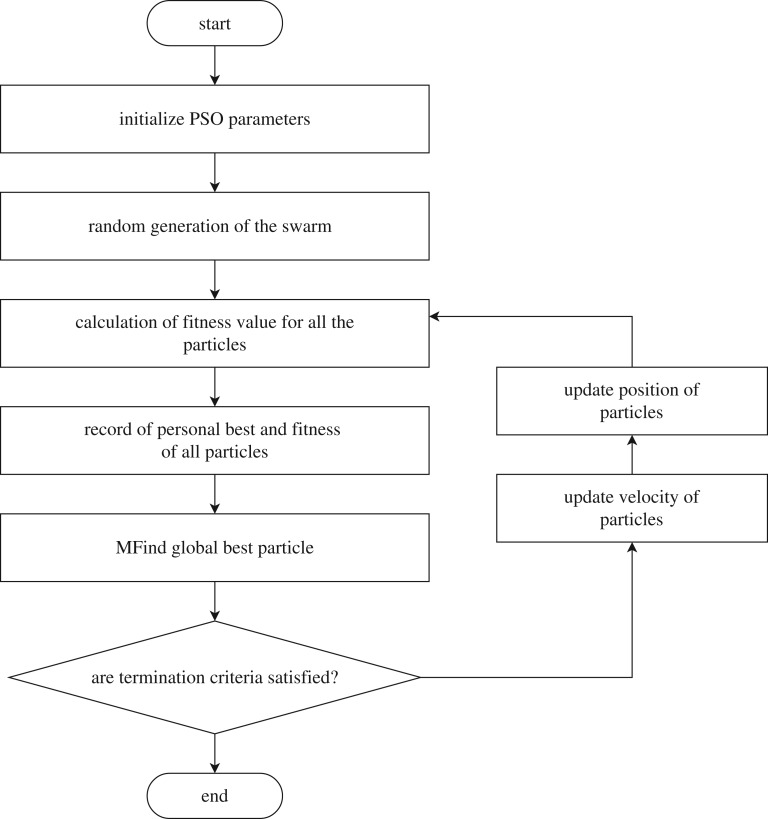
Flowchart of typical implementation of PSO algorithm.

In the PSO algorithm, with flowchart given in figure 8, each particle moves around in the *n*-dimensional space with a velocity (or accelerating) that is updated by *p*_best_ and the *g*_best_ position of the particle at each time step. The current position and the velocity of each particle are modified by the distance between its current position and *p*_best_, and the distance between its current position and *g*_best_ as given in the following. At each step *n*, by using the individual best position *p*_best_ and global best position *g*_best_, a new velocity for the *i*th particle can be modelled according to the following equation:

3.5 $$\begin{array}{rl} V_{i}(n) & =\chi [V_{i}(n-1)+\varphi_{1}r_{1}(p_{{\mathrm{best}}_{i}}-P_{i}(n-1))\\ & \;+\varphi_{2}r_{2}(g_{\mathrm{best}}-P_{i}(n-1))], \end{array}$$

where each particle represents a potential solution and it has a position represented by the position vector *P*_*i*_, with *r*_1_ and *r*_2_ denoting random numbers between 0 and 1; φ_1_ and φ_2_ being positive constant learning rates and *χ* is called the constriction factor defined as

3.6 $$\chi =\frac{2}{\left|2-\varphi -\sqrt{\varphi^{2}-4\varphi }\right|}\;\mathrm{and}\;\varphi =\varphi_{1}+\varphi_{2},\;\varphi >4.$$

Based on the updated velocity, each particle changes its position according to the following:

3.7 $$P_{i}(n)=P_{i}(n-1)+V_{i}(n).$$

The position is confined within the range of [*p*_min_, *p*_max_]. Changing position enables the *i*th particle to search around its local best position, *p*_best_, and global best position, *g*_best_.

PSO is an extremely simple algorithm that seems to be effective for optimizing a wide range of cost functions. The adjustment toward *p*_best_ and *g*_best_ by the optimizer is conceptually similar to the crossover operation used by genetic algorithms.

The PSO algorithm used in various optimization problems has certain advantages:

— it does not involve selection operation or mutation calculation, i.e. the search can be carried out by repeatedly varying particle’s speed;
— particles fly only to good areas, as learning from group’s experiences;
— it is based on artificial intelligence enabling broad application areas;
— it has a low computational cost.

Some of the disadvantages of the method may be listed as

— complexity increases exponential with the dimension of the optimization space;
— it is vulnerable to partial optimism, which leads to a sub-optimal regulation of its search speed and direction;
— with the lack of dimensionality this method cannot be used for problems of non-coordinate system, such as the solution to the energy field and the moving rules of the particles in the energy field.

To conclude, due to the flexibility and versatility of this algorithm, it can be used to overcome complex nonlinear optimization tasks like non-convex problems, being a good compromise between computational time and accuracy.

The function particleswarm has been implemented in Matlab with the following options:

— 10 independent calls of the algorithm;
— 1000 particles in the swarm;
— 500 maximum number of generations;
— 50 maximum stall iterations, to explore neighbourhood of a solution;
— 5 parameter to optimize;
— limited range; and
— 1 × 10^−9^ as limit value for the cost function minimum.

#### (iv) Optimization in feasible region

Irrespective of the optimization algorithm applied to nonlinear cost functions, there is no guarantee for convergence to the global minimum. The cost function defined for GA, PSO and the nonlinear least squares algorithm has been defined by using the so-called normalized mean square error, provided by the built-in Matlab function calNMSE. Its mathematical form is

3.8 $$\mathrm{NMSE}=\frac{||Z_{m}(j\omega )-Z_{e}(j\omega )|{|}^{2}}{||Z_{m}|{|}^{2}},$$

where *Z*_*m*_ is the measured impedance and *Z*_*e*_ the estimated impedance. The optimization algorithms use vector format and for this reason is useful to make another normalization by dividing the NMSE of (3.8) for the length of the impedance vector itself. This definition allows one to choose the cost function *J* as follows:

3.9 $$J=w_{r}\cdot {\mathrm{NMSE}}_{R}+w_{i}\cdot {\mathrm{NMSE}}_{I},$$

which is the weighted sum of the normalized mean square error for the real and the imaginary part of the impedance. The weight values *w*_*r*_ = 1 and *w*_*i*_ = 2 could be chosen differently. However, the problem is highly nonlinear in the five parameters and we propose to find the proper domain with nested loops, trying to optimize the cost function per decade of frequency. Monte Carlo analysis provided the empirical values for the upper and lower bounds in test sample groups (table 1).

**Table 1.** Upper and lower bounds for class NN1 (honey and glucose) and class NN2 (hand soap and shampoo) test samples.

class		*R*	*L*	*D*	*α*	*β*
NN1	min	0	−10^10^	10^8^	−2	0
	max	10^2^	−10^8^	10^10^	0	2
NN2	min	0	−10^5^	10^3^	−2	0
	max	10^2^	−10^3^	10^5^	0	2

## 4. Results

There are three identification methods applied to the experimental data in complex form. The comparison in terms of fitting performance did not deliver statistical meaningful differences. However, the CPU time evaluated for the total time of identification (including iterations) was

— genetic algorithms at 0.72 ± 0.26 s;
— PSO at 0.19 ± 0.13 s; and
— nonlinear least squares at 0.13 ± 0.05 s.

The identification was performed on a Dell OptiPlex 7060 desktop Intel Core I7 8th Gen, Win10 and Matlab R2017a version. Depending on the application, these times can be put into the context of continuous evaluation of context properties, in this case estimating the time-varying model parameters which may change over time. Given the ease of implementation and computational burden, the nonlinear least-squares algorithms seems to be the most practical.

**Figure 9.**
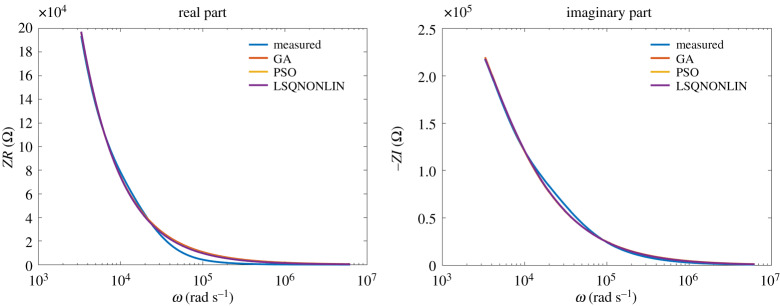
Glucose: comparison between optimization algorithms. (Online version in colour.)

**Figure 10.**
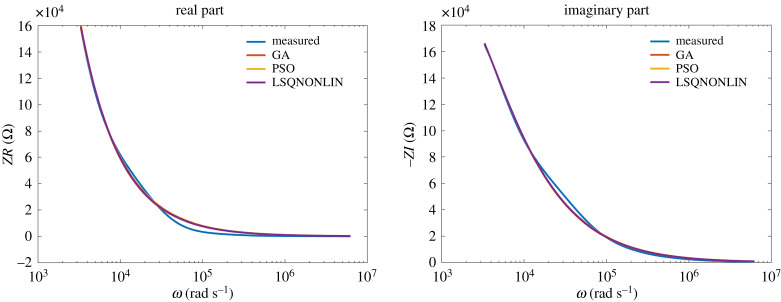
Honey: comparison between optimization algorithms. (Online version in colour.)

The model from (3.1) has been used to fit the experimental data. The results of the identification algorithms for class NN1 fluids are given in figures 9 and 10. It can be observed that the data are very well fitted by the model over many frequency decades.

**Figure 11.**
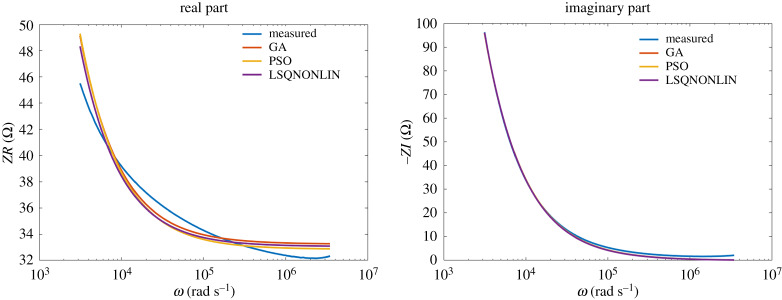
Hand soap: comparison between optimization algorithms. (Online version in colour.)

**Figure 12.**
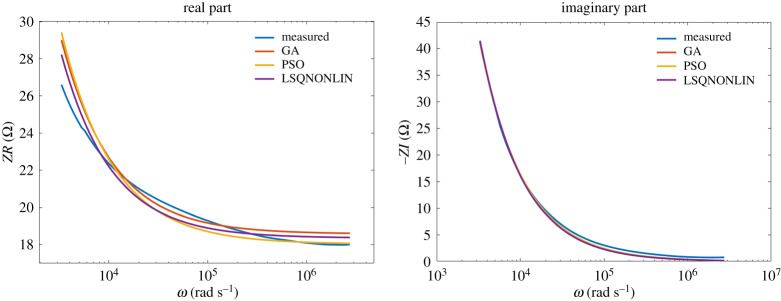
Shampoo: comparison between optimization algorithms. (Online version in colour.)

The results of the identification algorithms for class NN2 fluids are given in figures 11 and 12. Also in this case, the data are very well fitted by the model over many frequency decades.

No significant difference between the optimization algorithms was observed within the obtained results. This is an indication that the same (global) minimum was reached by all three algorithms. The model values are summarized in table 2.

**Table 2.** Identified model parameter values and normalized error.

		*R*	*L*	*D*	*α*	*β*	NMSE
glucose	GA	0	−5.35 × 10^9^	3.91 × 10^9^	−0.96	0.91	4.32 × 10^−5^
	PSO	0	−7.19 × 10^9^	5.84 × 10^9^	−0.95	0.92	4.46 × 10^−5^
	LSQ	0	−7.19 × 10^9^	5.84 × 10^9^	−0.95	0.92	4.28 × 10^−5^
honey	GA	0	−2.04 × 10^9^	3.61 × 10^8^	−1.10	0.82	4.53 × 10^−5^
	PSO	0	−3.26 × 10^9^	2.09 × 10^9^	−0.97	0.90	4.03 × 10^−5^
	LSQ	0	−5.05 × 10^9^	5.05 × 10^9^	−0.84	0.84	1.24 × 10^−4^
hand soap	GA	33.2	−1.67 × 10^4^	1.48 × 10^5^	−0.89	0.89	2.22 × 10^−5^
	PSO	32.5	−1.01 × 10^4^	1.37 × 10^5^	−0.89	0.89	2.16 × 10^−5^
	LSQ	32.8	−1.30 × 10^4^	2.66 × 10^5^	−0.89	0.89	2.22 × 10^−5^
shampoo	GA	18.5	−1.00 × 10^4^	3.92 × 10^4^	−2.00	0.84	6.46 × 10^−5^
	PSO	18.0	−1.00 × 10^4^	3.47 × 10^4^	−2.00	0.82	5.67 × 10^−5^
	LSQ	18.3	−1.03 × 10^4^	5.25 × 10^4^	−0.85	0.85	6.67 × 10^−5^

For reasons of ease of implementation, the nonlinear least-squares optimization method was used to fit the data presented hereafter. Another set of shear-thickening and shear-thinning NN fluids was tested to see whether the model parameter values was sensitive to variations in temperature, i.e. engine oil. The results are given in table 3. A non-monotonic evolution of the parameter values can be observed, which is expected from the shear-variations from figure 3.

**Table 3.** Identified model parameter values in engine oil as a function of temperature.

temperature	*R* × 10^3^	*L* × 10^9^	*D* × 10^9^	*α*	*β*
22^°^C	1.602	−2.124	7.099	−1.340	1.070
27^°^C	1.073	−3.041	6.584	−1.005	1.004
46^°^C	1.792	−1.955	7.008	−1.320	1.066
63^°^C	1.347	−2.152	8.099	−1.304	1.084

A set of NN fluids whose consistency is significantly different was tested to see whether the model parameter values was sufficiently able to distinguish among them. There are three test samples of food oils with results given in table 4. The results suggest the scaling factor *R* is proportional to the increase in consistency.

**Table 4.** Identified model parameter values in various food oils.

oil type	*R* × 10^3^	*L* × 10^9^	*D* × 10^9^	*α*	*β*
avocado	1.103	−5.146	7.271	−1.095	1.037
corn	4.543	3.422	2.934	−1.583	0.995
olive	6.691	−5.230	3.003	−1.576	0.997

Another set of NN fluids whose consistency varies was tested. The results for household fluids are given in table 5. Again, the results suggest the scaling factor *R* is proportional to the increase in consistency, a property of shear-thinning NN fluids.

**Table 5.** Identified model parameter values in household fluids.

type	*R* × 10^3^	*L* × 10^5^	*D* × 10^4^	*α*	*β*
soft detergent	2.671	−0.510	0.521	−0.818	0.827
hand soap	2.322	−0.507	0.516	−0.601	0.637
shampoo	3.514	−0.564	0.504	−0.628	0.651
standard detergent	4.415	−0.508	0.500	−0.755	0.787

An interesting set of NN fluid was that of thixotropic fluids, which resembles biological tissue. This was achieved by standard gelatin–water proportions, as given in increasing density in table 6. As expected from our prior expertise in lung tissue, the parameters *D* and *β* were most correlated to the change in viscoelastic properties of the sample. The compliance property, determined by this parameter as explained in [15], is consistently identified to decrease as the sample becomes more stiff.

**Table 6.** Identified model parameter values in mimicked biotissue consistency.

type	*R* × 10^3^	*L* × 10^9^	*D* × 10^9^	*α*	*β*
gelatin1	3.415	−0.510	0.604	−0.131	0.380
gelatin2	3.221	−0.486	0.501	−0.142	0.493
gelatin3	2.915	−0.681	0.320	−0.158	0.560
gelatin4	2.704	−0.690	0.310	−0.163	0.602

## 5. Discussion

The FOIM discussed in this paper was successfully employed in a prior study to fit the impedance of water–glucose solutions [34]. In this study, the parameters have quite small values, in the order of unity for constants, while *α* and *β* are in the order of 10^−3^ and 10^−4^, respectively. This is an indication that the solutions have properties closer to Newtonian fluids.

The FOIM and a simplified variant was used to identify honey and glucose properties in [35]. The simplified FOIM was in the form $R+D/s^{\beta }$ as the values of the *α* parameter were always negative. This observation is similar to the findings in this study as reported in table 2. Our findings are along the same lines as those in [36], where a FOIM variant was linked also to materials with viscoelastic properties.

For all sample tests, it seems that parameters *R* and *D* are most correlated to changes in viscosity. This is not at all surprising, as in [4,15], it was physically shown that a ladder network of *RC*-cells leads to appearance of fractional-order terms in this form in the limit impedance value, and that its value depends directly on the compliance represented by these cells. The application on lung tissue property modelling and identification from real data validated this theory.

In our study, the specific property of a NN fluid to demand fractional derivative order $Ls^{\alpha }$ was not found. We conclude that other types of fluids with properties exhibiting increasing high-frequency dependence will require this term.

The relevance of this work is substantial in its fundamental nature as identification for control in NN fluid dynamic environment has quite a large number of cross-disciplinary applications.

For instance, some applications of modelling NN fluids are

— prediction of glacier movement (mixture of ice+water) [37]
— prediction of blood properties as a result of medication and or arterial disease progress [5]
— evolution and dynamics of ground (muddy) water transportation systems [38].

Some applications of (model based) control are

— positioning particles in electro-magnetic actuated fluids (e.g. in liquid steel manufacturing in the continuous casting process) [39]
— guiding nano-sensors for detection of structural changes in blood arterial walls [3,8,40]
— navigating via servocontrol the unmanned underwater vehicles in water+ice mixtures to investigate climate change variations and/or geological changes [41].

A limitation of this study is that the proposed model, despite its versatility, was not perfectly able to fit the data on large range of frequencies. Some NN fluids, such as engine oils depend on temperature in a non-monotonic progression, and some frequency intervals are better fit than others. Such non-local elastic properties are visible in materials under mechanical and momentum stress [42]. This observation may suggest that other models could be used in those particular frequency intervals. Although several variants of FOIM exist, the choice of the frequency interval and model structure depends heavily on the end-objective of the identification exercise and application use.

## 6. Conclusion

The paper presents a minimal parameter versatile FOIM to identify viscosity-related properties in NN fluids. The experimental test samples and identified model parameters suggest the model is adequate to determine variations in fluid properties from several applications.

## Supplementary Material

Data results

## Supplementary Material

Data appendix

## Data accessibility

Supporting data have been provided as electronic supplementary material along with this article.
