# Supplementary material for: Mathematical modelling with experimental validation of viscoelastic properties in non-Newtonian fluids
Source: Philos Trans A Math Phys Eng Sci. 2020 May 11;378(2172):20190284. doi: 10.1098/rsta.2019.0284 (PMC7287316; doi:10.1098/rsta.2019.0284)
Supplement: Data results [file rsta20190284supp1.zip › data_results/Supplementary material appendix results.pdf]

Mathematical models with experimental validation of Non-Newtonian fluid properties

C. Ionescu, I. Birs, D. Copot, C. Muresan, R. Caponetto

Philosophical Transactions A

DOI: 10.1098/rsta.2019.0284

Supplementary Figures 9-12.

the following steps have to be performed in order to run the matlab code to obtain the fitting results

create the following m. files by copying the following codes ***callNMSE ; data_managing; err_fun and error_fun*** to matlab script

create a main file with the ***Nlsq_RLD*** to perform the identification

callNMSE

```
%% Copyright(c) Naushad Ansari, 2017.
% %% Please feel free to use this open-source code for research purposes only.
% %%
% %% contact at naushadansari09797@gmail.com in case of any query.
% %%
% %%
% %% This function calculates the nmse of a signal with reference to original
% signal. NMSE can be calculated for 1-D/2-D/3-D signals.
%%---------------------------------------------------------------------%%
%%---------------------------------------------------------------------%%
% %% output: nmse-> nmse (normalized mean square error)
%
% %% input:  orgSig-> original 1-D/2-D/3-D signal (or reference signal)
%            recSig-> reconstructed (1-D/2-D/3-D) signal/ signal obtained
%            from the experiment/ signal, of which nmse is to be calculated
%            with reference to original signal.
%            boun-> boun is the boundary left at the corners for the
%            nmse calculation.  default value = 0
%%---------------------------------------------------------------------%%
%%---------------------------------------------------------------------%%
function nmse=calNMSE(orgSig,recSig,varargin)

if isempty(varargin)
    boun = 0;
else boun = varargin{1};
end

if size(orgSig,2)==1       % if signal is 1-D
    orgSig = orgSig(boun+1:end-boun,:);
    recSig = recSig(boun+1:end-boun,:);
else                       % if signal is 2-D or 3-D
    orgSig = orgSig(boun+1:end-boun,boun+1:end-boun,:);
```

```
    recSig = recSig(boun+1:end-boun,boun+1:end-boun,:);
end

mse=norm(orgSig(:)-recSig(:),2)^2/length(orgSig(:));
sigEner=norm(orgSig(:))^2;
nmse=(mse/sigEner);
```

data_managing

```
function [fr,w,mod,ph,re,im] = data_managing(fname, cut_signal, w_start, w_stop)
%fname='honey';

load([fname,'.mat'])

if(cut_signal)
    if(w_stop)
        cut_ind = find(omega > w_stop);%finding elements which are greater than w_stop
        cut_ind(2:end)=[];
        f(cut_ind:end)=[];
        omega(cut_ind:end)=[];
        Z_mod(cut_ind:end)=[];
        Z_ph(cut_ind:end)=[];
        ZR(cut_ind:end)=[];
        ZI(cut_ind:end)=[];
    end
    if(w_start)
        cut_ind = find(omega > w_start);
        cut_ind(2:end)=[];
        f(1:cut_ind)=[];
        omega(1:cut_ind)=[];
        Z_mod(1:cut_ind)=[];
        Z_ph(1:cut_ind)=[];
        ZR(1:cut_ind)=[];
        ZI(1:cut_ind)=[];
    end
end

fr=f;
w=omega;
mod=Z_mod;
```

```
ph=Z_ph;
re=ZR;
im = ZI;
end
```

err_fun

```
function E = err_fun(x)

global Z_meas omega

Z_est = x(1)+x(2)*(1i*omega).^(x(4))+x(3)*(1i*omega).^(-x(5));

E = [real(Z_meas - Z_est); imag(Z_meas - Z_est)];

end
```

error_fun

```
function out = error_fun(x)

global omega ZR ZI

% load([fname,'.mat'])
%
%  omega(1:cut_ind)=[];
%  ZI(1:cut_ind)=[];
%  ZR(1:cut_ind)=[];

Z_est = x(1)+x(2)*(1i*omega).^(x(4))+x(3)*(1i*omega).^(-x(5));

re_err = calNMSE(ZR,real(Z_est));
im_err = calNMSE(ZI,imag(Z_est));

out = re_err + 2*im_err;

end
```

Nlsq_RLD

```
clc; clear all; close all; clear memory

%% load data
Data_NN1=readtable('Data_NN1.txt');

f=Data_NN1.f; omega=Data_NN1.omega; ZI=Data_NN1.ZI; ZR=Data_NN1.ZR;
Z_mod=Data_NN1.Z_mod; Z_ph=Data_NN1.Z_ph;
save  ('dataNN1.mat', 'f', 'omega', 'ZI', 'ZR', 'Z_mod', 'Z_ph')
%%
t_start = tic;
global omega Z_meas ZR ZI

fname='dataNN1'
cut_signal = 1; %Boolean flag: 1-> cut signal, 0-> keep original signal
w_start =628; %Starting frequency in [rad/s]; put 0 if no initial cut is needed
w_stop  = 000000; %Ending frequency in [rad/s]; put 0 if no final cut is needed
n = 10; %Number of different calls of the optimization algorithms



Rd =[0;1e8]; Ld =[-1e10;-1e2]; Dd =[1e4;1e10]; ad =[-2; 1]; bd =[0.1;2];
dom =[Rd,Ld,Dd,ad,bd];

%%  Data Managing
[f,omega,Z_mod,Z_ph,ZR,ZI] = data_managing(fname,cut_signal,w_start,w_stop);
Z_meas = ZR+(1i*ZI);

%% PSO

tic;

str_pso = {'Particle Swarm Optimization'};
p_pso = [];
```

```
%n° of iteration 500 while by default it is set to 1000

opt =
optimoptions('particleswarm','SwarmSize',1000,'MaxIterations',500,'MaxStallIterations',50,'Hybrid
Fcn',@patternsearch,'UseParallel',false,'FunctionTolerance',1e-7,'Display','off');


for k= 1:n                    %20 different calls of PSO algorithm

    [param,nmse,exitflag,output] = particleswarm(@error_fun,5,dom(1,:),dom(2,:),opt);

    p_pso = [p_pso;[param,nmse,exitflag]];

end


[min_err_pso,ind_pso] = min(p_pso(:,6));

best_values_pso = p_pso(ind_pso,1:5);


Z_est_pso = best_values_pso(1)+best_values_pso(2)*(1i*omega).^(best_values_pso(4)) +
best_values_pso(3)*(1i*omega).^(-best_values_pso(5));


duration_pso = toc/60;


%% LSQNONLIN

tic;

p_lsq = [];

for k = 1:1000

    init = [16000 -10 10 -.05 0.09];

    lsq_opt = optimoptions(@lsqnonlin,'MaxIterations',500,'FunctionTolerance',1e-
9,'Display','none');

    param = lsqnonlin(@err_fun,init,dom(1,:),dom(2,:),lsq_opt);

    ztemp = param(1)+param(2)*(1i*omega).^(param(4)) + param(3)*(1i*omega).^(-param(5));

    error = calNMSE(ZR,real(ztemp)) + 2*calNMSE(ZI,imag(ztemp));

    p_lsq = [p_lsq; [param,error,init]];

end

[min_err_lsq,ind_lsq] = min(p_lsq(:,6));

best_values_lsq = p_lsq(ind_lsq,1:5);

init = best_values_lsq;

for k = 1:n
```

```
    lsq_opt = optimoptions(@lsqnonlin,'MaxIterations',500,'FunctionTolerance',1e-9,'Display','off');
    param= lsqnonlin(@err_fun,init,dom(1,:),dom(2,:),lsq_opt);
    ztemp = param(1)+param(2)*(1i*omega).^(param(4)) + param(3)*(1i*omega).^(-param(5));
    error = calNMSE(ZR,real(ztemp)) + 2*calNMSE(ZI,imag(ztemp));
    if(error < min_err_lsq)
        init = param;
    end
end
min_err_lsq = error;
best_values_lsq = param;
Z_est_lsq = best_values_lsq(1)+best_values_lsq(2)*(1i*omega).^(best_values_lsq(4)) +
best_values_lsq(3)*(1i*omega).^(-best_values_lsq(5));
duration_lsq = toc/60;

%% PLOT Results
 full = [370 140 670 460];   % suitable figure dimension to get png/eps(in 1920x1280 screen)
figure%('Name','Amplitude Meas vs Est','Position',full);
semilogx(omega,20*log10(Z_mod),omega,20*log10(abs(Z_est_pso)),omega,20*log10(abs(Z_est
_lsq)),'LineWidth',2)
ylabel('Amplitude [dB]','FontSize',14,'FontWeight','bold')
xlabel('\omega [rad/s]','FontSize',14,'FontWeight','bold')
title('Bode Amplitude','FontSize',15)

figure('Name','Phase Meas vs Est','Position',full);
semilogx(omega,Z_ph,omega,rad2deg(angle(Z_est_pso)),omega,rad2deg(angle(Z_est_lsq)),'Lin
eWidth',2)
ylabel('Phase [°]','FontSize',14,'FontWeight','bold')
xlabel('\omega [rad/s]','FontSize',14,'FontWeight','bold')
title('Bode Phase','FontSize',15)

figure('Name','Real Part Meas vs Est','Position',full);
semilogx(omega,ZR,omega,real(Z_est_pso),omega,real(Z_est_lsq),'LineWidth',2)
ylabel('ZR  [\Omega]','FontSize',14,'FontWeight','bold')
xlabel('\omega [rad/s]','FontSize',14,'FontWeight','bold')
```

```
title('Real Part','FontSize',15)

figure('Name','Imaginary Part Meas vs Est','Position',full);
semilogx(omega,-ZI,omega,-imag(Z_est_pso),omega,-imag(Z_est_lsq),'LineWidth',2)
ylabel('-ZI  [\Omega]','FontSize',14,'FontWeight','bold')
xlabel('\omega [rad/s]','FontSize',14,'FontWeight','bold')
title('Imaginary Part','FontSize',15)
```
